# Non-Autoimmune Hemolytic Anemia with Clostridium Ramosum Bacteremia

**DOI:** 10.4137/ccrep.s763

**Published:** 2008-05-22

**Authors:** Aref Al-kali, Alok Oswal, Arafat Tfayli

**Affiliations:** 1 Hematology and Oncology Section, Oklahoma University, 920 Stanton Young Blvd, WP 2040, Oklahoma City, OK 73104.; 2 Internal Medicine Department, Oklahoma University, 920 Stanton Young Blvd, WP 1160, Oklahoma City, OK 73104.; 3 Hematology and Oncology Section, Fellowship Director, Oklahoma University, 920 Stanton Young Blvd, WP 2040, Oklahoma City, OK 73104.

## Introduction

Clostridium Ramosum is an intestinal anaerobe first isolated from a patient with pulmonary gangrene and appendicitis by Veilon and Zuber in 1898.^1^ It is usually present in normal fecal flora of man but has been found in association with osteomyelitis, liver abscess, mastoiditis, otitis, cerebellar and lung abscesses.^2^,^3^,^4^,^5^ This is the first case report of hemolytic anemia caused by Clostridium Ramosum in an elderly immunocompromised patient.

## Case Report

A 60 year old white male with a history of relapsed acute myeloid leukemia (AML) presented in May 2007 to our university hospital with complaints of fever and headache for few days prior to admission. He was diagnosed with AML type M1 (by French-American-British classification) in February 2005. He received induction with “7 + 3” (cytarabine and idarubicin) followed by high dose ARA-C consolidation. He stayed in complete remission until December 2006 when his leukemia relapsed and was re-induced with the Flag-Ida regimen. Patient then underwent non-myeloablative allogeneic Stem Cell Transplantation from an unrelated donor in December 2006. The leukemia unfortunately relapsed again in April 2007 and the patient was treated with single agent Gemtuzumab (Mylotarg) for two doses 2 weeks apart. He was re-admitted in May 2007 for neutropenic fever and a headache which had been ongoing for approximately three days. Examination revealed no abnormal findings except for a 4 cm vesicular skin lesion on his back. He was empirically started on acyclovir for possible zoster infection, as well as vancomycin and ceftazidime for coverage of neutropenic fever. Laboratory investigations on admission revealed hemoglobin of 8.9 g/dl, platelet count of 2,000/mm^3^, and white blood count (WBC) of 200/mm^3^. His hemoglobin continued to fall despite repeated blood transfusions down to 7.9 g/dl (Table 1). Work-up for the cause of anemia revealed negative stool guaiac, disseminated intravascular coagulation (DIC) panel, and Coomb’s test but an elevated lactic dehydrogenase (LDH) (1840 u/L), and a low haptoglobin (33 mg/dl). Peripheral blood smear showed severe pancytopenia, with no evidence of schistocytes or blasts. The elevated LDH and decreased haptoglobin pointed towards this being hemolytic in nature. Bone marrow biopsy revealed persistent myeloblasts. CT scan of the head, chest x-ray, lumbar puncture, and urine analysis were all negative. Serum *aspergillus* and *cytomegalovirus* antigenemias were both negative. Other infectious etiologies like *parvovirus* and *malaria* were not ruled out. Two blood cultures isolated *c. ramosum*. Based on cultures and sensitivities, the patient was switched to Ampicillin/Sulbactam (Unasyn). After a few days, the patient’s condition improved. His hemolysis improved proven by decreased LDH to 1144. The LDH did not completely normalize with therapy of the infection, presumably because the patient was requiring frequent red blood cell (RBC) transfusions causing a persistently elevated LDH. It appeared that his hemolysis was indeed secondary to septicemia caused by this pathogen. At same time, he developed mild diarrhea and a stool sample for *clostridium difficile* was positive for the antigen but negative for toxin B. It was presumed to be a cross-reaction between the two *clostridium* species, *ramosum* and *difficile*. This proved to be true when his diarrhea subsequently resolved without further treatment. Patient kept getting packed RBC transfusions as needed secondary to persistent AML but at a lower frequency. He actually got nine units during the first ten days of admission, but then the rate of transfusion dropped to two units every ten days by the time he was discharged. The rate of platelets transfusions didn’t improve.

*Clostridium ramosum* is an anaerobe commonly found in the human intestinal tract. It has been associated with several clinical conditions thus far but has never been correlated with hemolytic anemia. This should be especially suspected if a sudden increase in transfusion requirements with an increase of LDH level and a decrease in haptoglobin level happen. Associated fever would raise the suspicion of an infectious etiology behind this. There have been several case reports that documented severe hemolytic anemias with *c. perfringens*. This is thought to be secondary to alpha-toxins. *C. ramosum* is known to produce an IgA proteinase that superficially cleaves human IgA1 and IgA2m allotype.^6^ Whether this has any correlation with a hemolytic process has not been reported yet. *C. ramosum* has been implicated in more aggressive infections lately, especially with abscesses and deep tissue infections.^4^

## Conclusion

This is the first case report of a hemolytic anemia being caused secondary to *c. ramosum* septicemia which was effectively treated with Unasyn. Early recognition of this condition led to an effective treatment of the infection and the subsequent hemolytic anemia. LDH and the frequency of transfusions can serve as a marker of improvement.

## Figures and Tables

**Table 1:** Blood Work-up during hospitalization.

	Blood cultures	Hemoglobin	LDH
Day **1**(5/11/7)	C. Ramosum	8.9	1629
Day **3**	Neg	8.8*	1298
Day **6**	Neg	8.1*	1144
Day **14**	Neg	7.9*	1520
Day **24**	Neg	8.2*	1247

* After transfusion.
